# Inverse tuning of metal binding affinity and protein stability by altering charged coordination residues in designed calcium binding proteins

**DOI:** 10.1186/1757-5036-2-11

**Published:** 2009-12-21

**Authors:** Anna Wilkins Maniccia, Wei Yang, Julian A Johnson, Shunyi Li, Harianto Tjong, Huan-Xiang Zhou, Lev A Shaket, Jenny J Yang

**Affiliations:** 1 Department of Chemistry, Center for Drug Design and Biotechnology, Georgia State University, Atlanta, GA 30303, USA; 2 Changchun Institute of Applied Chemistry, Chinese Academy of Sciences, Renmin Road 5625, Changchun, Jilin 130022, PR China; 3 Department of Physics and Institute of Molecular Biophysics and School of Computational Science, Florida State University, Tallahassee, Florida 32306, USA

## Abstract

Ca^2+ ^binding proteins are essential for regulating the role of Ca^2+ ^in cell signaling and maintaining Ca^2+ ^homeostasis. Negatively charged residues such as Asp and Glu are often found in Ca^2+ ^binding proteins and are known to influence Ca^2+ ^binding affinity and protein stability. In this paper, we report a systematic investigation of the role of local charge number and type of coordination residues in Ca^2+ ^binding and protein stability using *de novo *designed Ca^2+ ^binding proteins. The approach of *de novo *design was chosen to avoid the complications of cooperative binding and Ca^2+^-induced conformational change associated with natural proteins. We show that when the number of negatively charged coordination residues increased from 2 to 5 in a relatively restricted Ca^2+^-binding site, Ca^2+ ^binding affinities increased by more than 3 orders of magnitude and metal selectivity for trivalent Ln^3+ ^over divalent Ca^2+ ^increased by more than 100-fold. Additionally, the thermal transition temperatures of the apo forms of the designed proteins decreased due to charge repulsion at the Ca^2+ ^binding pocket. The thermal stability of the proteins was regained upon Ca^2+ ^and Ln^3+ ^binding to the designed Ca^2+ ^binding pocket. We therefore observe a striking tradeoff between Ca^2+^/Ln^3+ ^affinity and protein stability when the net charge of the coordination residues is varied. Our study has strong implications for understanding and predicting Ca^2+^-conferred thermal stabilization of natural Ca^2+ ^binding proteins as well as for designing novel metalloproteins with tunable Ca^2+ ^and Ln^3+ ^binding affinity and selectivity.

**PACS codes: **05.10.-a

## 1. Background

Electrostatic interactions have been shown to be important for many biological processes [1]. Additionally, they are known to be important for the stability and folding of proteins, though their net effects can vary greatly from protein to protein, depending on the relative contributions of direct Coulomb interactions between charges and the desolvation cost [2]. A series of studies on surface charge variants of a cold shock protein has revealed that the context of mutations rather than the net protein charge is a critical determinant of protein stability [3-5]. If net charge were an important factor, one would expect proteins with small net charges to be more stable than proteins with large net charges. However, Henzl and Graham reported that β-parvalbumin, with a net charge of -16, displays a 7°C higher thermal transition temperature than a close homologue, α-parvalbumin, with a net charge of -5 [6,7].

In contrast to the variable effects of charged surface residues, Ca^2+^-binding to charged coordination residues almost always increases protein stability, simply due to selective ion binding to the folded state over the denatured state [8]. In the absence of Ca^2+^, calmodulin (CaM) is highly dynamic and labile even at room temperature [9,10]. Upon cooperatively binding four Ca^2+ ^ions, the *T*_m _of CaM increased to over 90°C. Additionally, proteins such as thermolysin, α-amylase, cadherin, subtilisin, and protein S bind Ca^2+ ^to enhance thermal and proteolytic stability [11-14]. Many of the recent advances in the development of thermally stable enzymes for industrial use require the use of Ca^2+ ^for stability [12,15].

In addition to their effect on thermal stability, electrostatic interactions contribute greatly to Ca^2+^-binding affinity [16-19]. A survey has shown that 106 of 165 EF-hand Ca^2+ ^binding sites contain 4 negatively charged coordination residues [20]. Our recent comprehensive analysis of the available structures in the Protein Data Bank also reveals that three and four negative charges are frequently observed in the coordination shells of natural Ca^2+^-binding sites [21]. In general, removal of charged coordination residues is found to reduce Ca^2+^-binding affinity, though theories about the role of charge effects in Ca^2+^-binding affinity [8,16], such as the acid-pair hypothesis [18], are often inconsistent with experimental observations of Ca^2+ ^binding affinity in mutant Ca^2+^-binding proteins, such as EF-hand proteins [22,23]. Replacements of Asp and Glu at the conserved loop positions 1 and 12 with non-charged residues significantly decreased Ca^2+ ^binding affinity of several EF-hand proteins [24]. Additionally an Asp-to-Asn mutation at Site III of CaM led to a decrease in both Ca^2+ ^binding affinity and cooperativity at the C-terminal domain [16,25]. On the other hand, the same mutation at Site II of CaM led to the opposite effect at the N-terminal domain. Henzl *et al*. have shown that increasing the net negative charge in the coordination sphere of rat α- and β-parvalbumins from -4 to -5 increased Ca^2+ ^binding affinity [26,27], while Falke *et al*. have shown that in galactose-binding protein, increasing the net negative charge from -3 to -4 decreased affinity for Ca^2+ ^but increased affinity for trivalent metal ions [28]. Factors that may explain the variations in observed effects of charge mutations on metal-binding affinity include cooperation between different metal-binding sites on the same protein, conformational entropy, and contribution of the protein environment. To separate out the contribution of charged coordination residues to Ca^2+ ^binding affinity, extensive work has also been conducted utilizing peptide fragments encompassing the Ca^2+ ^binding sites of several proteins. Unfortunately, this approach suffers from the limitations of ill-defined structures in solution and peptide aggregation [8,16,29-31]. To date, a quantitative description of local charge effects on Ca^2+ ^binding and stability has not been established.

**Figure 1:**
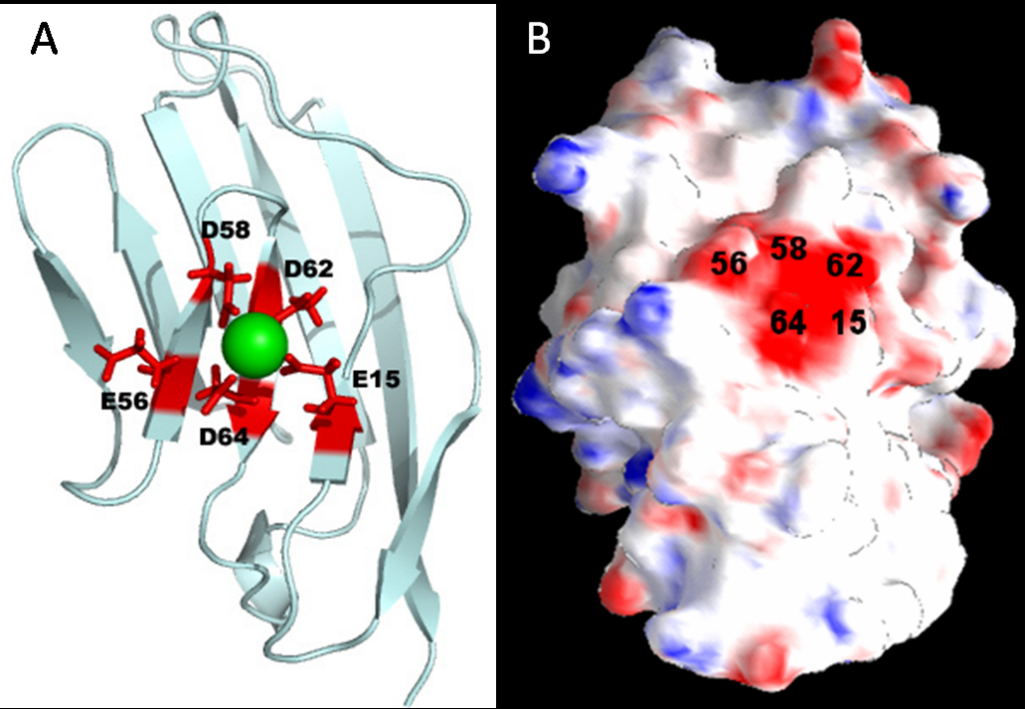
**Model structure of designed proteins**. (A) Model structure of EEDDD (7E15) and the coordination design of the variants. (B) The surface potential of Ca^2+^-free EEDDE, calculated using DelPhi[45], shows the highly-charged Ca^2+^-binding location. The calculation used interior and exterior dielectric constants of 4 and 80, respectively.

We have developed a design strategy to overcome the confounding factors noted above, allowing for a comprehensive study of local determinants for Ca^2+ ^binding affinity and thermal stability [8,19,32]. Our first designed Ca^2+^-binding protein did not possess a stable structure at room temperature [8]. However, since then, several proteins with stable structures have been designed and engineered. Among them, CD2.trigger undergoes Ca^2+^-dependent conformational change (unpublished data) while 7E15 [32], 6D15 (also termed Ca.CD2) [19], and 6D79 [33] do not exhibit global conformational changes upon Ca^2+ ^binding. These designed proteins are excellent model systems for gaining insight into the tradeoff between Ca^2+ ^binding affinity and protein stability without the limitations associated with natural Ca^2+^-binding proteins [32]. In this paper, we report our systematic investigation of the roles of local charge and coordination residue type on Ca^2+ ^binding affinity and Ca^2+^-conferred thermal stabilization. To investigate the effects of local charges without the interference of protein global conformational change, CD2.7E15 (Fig. 1) was chosen as the template, primarily because it tolerates up to five negative charges at the binding site instead of four negative charges as in 6D15 and 6D79 [32,33]. Additionally, CD2.7E15 retains a CD2-like fold and no significant conformational changes are observed due to binding of Ca^2+ ^(*K*_d _~ 0.1 mM) in the designed pocket. Using various biophysical measurements as well as theoretical calculations, we show that, when the number of charged coordination residues increases from 2 to 5, Ca^2+ ^binding affinities and metal selectivity for trivalent Ln^3+ ^over divalent Ca^2+ ^increases, while the thermal transition temperatures of the proteins in the absence of cations decrease. Ca^2+ ^or Ln^3+ ^binding to the designed Ca^2+^-binding pocket, however, allows the proteins to regain thermal stability. The results suggest that in a relatively restricted Ca^2+^-binding site, more negative charges facilitate binding of Ca^2+ ^and Ln^3+ ^accompanied by a tradeoff in protein stability due to significant repulsion among the negatively charged coordination residues.

## 2. Methods

### 2.1. Protein engineering and purification

CD2.7E15 (also referred to as EEDDD in this study) variants were engineered using the classical PCR method from CD2.7E15 DNA in the vector pGEX-2T. The mutations were confirmed using automated DNA sequencing at the Biology Core Facility of Georgia State University. The protein expression, purification, and concentration estimation were carried out as previously reported [32]. The background Ca^2+ ^was minimized by incubating the sample with EGTA first, followed by a pH gradient separation using a HiTrap SP HP column (GE Healthcare) with chelexed (Chelex-100 resin, BioRad) buffers.

### 2.2. Trp fluorescence

Trp emission spectra over 300-400 nm, with excitation at 282 nm, of proteins (4 μM) in 10 mM Tris, pH 7.3 in the presence of 10 mM Ca^2+ ^or 1 mM EGTA were collected using a PTI fluorimeter and a cuvette with a 1 cm pathlength at room temperature.

### 2.3. Metal binding

Cation binding was done in 20 mM PIPES-10 mM KCl, pH 6.8. The Tb^3+^-FRET fluorescence signals were monitored using a PTI fluorimeter following the procedure described previously. The Tb^3+ ^binding affinity was measured by direct Tb^3+ ^titration and the La^3+ ^binding affinity was derived using the competitive binding of La^3+ ^against Tb^3+^. The Ca^2+^-binding affinity was measured using ^1^H-^15^N HSQC NMR spectra by titrating Ca^2+ ^into the protein sample with 40-50 μM EGTA at the initial point and monitoring the chemical shift changes versus the Ca^2+ ^concentrations. The *K*_d _of each CD2.7E15 variant was calculated using the average of results from multiple resonances and the uncertainty represented the different responses of these resonances to Ca^2+ ^binding.

### 2.4. Far-UV CD

Far-UV CD spectra were collected on a Jasco-810 spectropolarimeter coupled with a Peltier temperature controller. The signal was monitored over 260-200 nm with four repeat scans. The proteins (25 μM) were in 10 mM Tris-10 mM KCl, pH 7.3 with either 1 mM EGTA, 10 mM Ca^2+^, or 0.05 mM Tb^3+^, placed in a 1 mm pathlength cell with a sealed top. Buffer signals were subtracted from the spectra. For thermal titration measurements, CD spectra were taken at 2-5 degree increments with four to six repeat scans over a temperature range from 15 to 85°C. Five minutes were allowed for equilibration at each temperature before the scans were taken. The signal change at 225 nm was plotted using KaleidaGraph and fitted using a two-state transition model,(1)

where Δ*S *and Δ*S*_max _are the signal changes at temperature *T *point and the final temperature, respectively; *T*_m _is the transition temperature; and *k *is a fitting parameter measuring the steepness of the thermal transition. The reported *T*_m _and the uncertainty are the average and standard deviation of three runs, respectively.

### 2.5. Prediction of mutational effects on folding stability and Ca^2+ ^binding affinity

The model structures of EEDDD with bound Ca^2+ ^were generated by the design program and the structures of the other variants were generated from EEDDD using the program SYBYL (Tripos Co.). Hydrogen atoms were added using SYBYL. The bound Ca^2+ ^was removed for the apo-form structures, and the Ca^2+ ^was replaced by a Tb^3+ ^at the same location to construct the Tb^3+^-loaded form structures. All Asp and Glu residues were assumed to be unprotonated (and all Lys and Arg protonated); while p*K*_a _values are prone to be perturbed when charges are clustered [34], the assumed protonation states seem appropriate for the neutral pH where melting temperatures and ion binding affinities were measured.

The Poisson-Boltzmann (PB) equation was solved to calculate the electrostatic free energies of the protein variants in bound- and apo-forms using the UHBD program[35]. A salt concentration of 10 mM in the solution was used. Electrostatic contributions of mutational effects on the Ca^2+ ^binding affinity and folding stability of CD2.7E15 variants were calculated following previously-published protocols [4,36]. The effect of a mutation on the folding stability was calculated as the change in the electrostatic folding free energy:(2)

In our case, the wild-type protein was identified as the original 7E15 (i.e., EEDDD), and the mutant was any of the variants introduced here. The unfolded state of proteins was modeled as individual residues separately dissolved in the solvent. Therefore, in the unfolded state, residues other than the one under mutation make the same contribution to the electrostatic free energies of the wild-type protein and mutant, and do not affect ΔΔ*G*_f_. Neglecting this contribution, the electrostatic folding free energy of either the wild-type protein or the mutant is:(3)

where *G*_el_(X) is the electrostatic free energy of molecule X; "protein" refers to the folded protein; and "residue" refers to a residue, the one under mutation, that is carved out of the folded structure. *G*_el _has both a Coulombic component and a solvation component.

Calculations of ΔΔ*G*_f _were done on the apo forms only. A basic assumption underlying the present study is that the metal ions bind to the designed proteins only when they are in the folded state, since only then coordination residues come together to form the binding site. Under this assumption, a state in which the protein is both unfolded and metal ion-loaded does not exist, and it would not be appropriate to apply the procedure for calculating ΔΔ*G*_f _to the metal ion-loaded forms. We note that preferential binding of the folded state by metal ions shifts the folding-unfolding equilibrium towards the former.

The contribution of a point mutation to metal ion binding affinity can be expressed as the binding free energy difference between the mutant and the wild-type complex:(4)

where Δ*G*_b _is the binding free energy, calculated as(5)

The calculated result is to be compared with the counterpart from experimentally determined binding affinity:(6)

where *k*_B _is Boltzmann's constant.

## 3. Results

### 3.1. Engineering of CD2.7E15 variants

In a previous publication [32], CD2.7E15 referred to a rationally designed Ca^2+^-binding protein with an engineered Ca^2+^-binding site at the B, E, and D β-strands of the host protein CD2 (Fig. 1), with coordination residues Glu, Glu, Asp, Asp, and Asp occupying positions 15, 56, 58, 62, and 64, respectively. Here CD2.7E15 is referred to as EEDDD; other variants of EEDDD are referred to using a similar notation, consisting of single-letter codes for the residues occupying the five coordination sites. The EEDDD variant was chosen as the template to investigate the effects of charge and coordination residue type on Ca^2+ ^binding based on the following considerations. First, this metal binding site presented the possibility to study the relationship between Ca^2+ ^binding and protein stability. We have previously shown that the cluster of five negatively charged residues at this location did not unfold the protein and metal binding did not alter the global conformation of the protein [32]. Second, this variant retains two wild-type residues, E56 and D62. Third, using the EEDDD template, a series of variants with local net charges ranging from -5 to -2 by replacing Asp or Glu with Asn or Gln (Table 1) could be generated. Variants with local net charges of -1 or 0 were not generated since they were not expected to bind Ca^2+ ^with a reasonable affinity. Finally, using the EEDDD template, pairs of variants, such as EEDDD/EEDDE or EEDDN/EEDDQ, which possess identical net charges but differ by one methylene group, could also be generated, presenting the opportunity to investigate the size effects of coordination residues.

**Table 1:** The cation binding affinities for 7E15 variants

Protein	Residues					Charge	*K*_d_		
			
	15	56	58	62	64		Tb^3+ ^(μM)^*a*^	La^3+ ^(μM)^*a*^	Ca^2+ ^(mM)^*b*^
CD2	N	E	L	D	K	-1	NB	NB	NB
EEDDD	E	E	D	D	D	-5	0.4 ± 0.2	0.5 ± 0.1	0.10 ± 0.05
EEDDE	E	E	D	D	E	-5	0.8 ± 0.2	0.7 ± 0.1	1.1 ± 0.4
EEDDN	E	E	D	D	N	-4	6.3 ± 0.6	2.2 ± 0.4	2.1 ± 0.5
EEDDQ	E	E	D	D	Q	-4	14 ± 3	3.2 ± 0.7	0.7 ± 0.2
EENDN	E	E	N	D	N	-3	> 30	> 15	> 10
NENDN	N	E	N	D	N	-2	NB	NB	NB

Buffer condition: 20 mM PIPES-10 mM KCl, pH 6.8. NB: No observed binding. *a*: from Tb^3+^-FRET. *b*: from NMR HSQC.

### 3.2. Conformational analysis of the 7E15 variants

**Figure 2:**
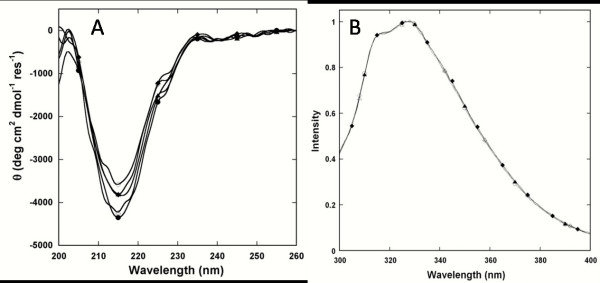
**Conformational analysis by far-UV-CD and Trp fluorescence**. (A) The far-UV CD spectra of all 7E15 variants in the presence of 1 mM EGTA or 10 mM Ca^2+ ^in 10 mM Tris, pH 7.4 show a single negative maximum at ~216 nm. CD2 with Ca^2+ ^(open circle), EEDDQ with EGTA (open diamond) or Ca^2+ ^(solid diamond), and EENDN with EGTA (open triangle) or Ca^2+ ^(solid triangle) are shown as examples. (B) The normalized Trp fluorescence spectra for all 7E15 variants almost overlap with a single emission maximum at 327 nm. CD2(open circle), EEDDN with EGTA (open diamond) or Ca^2+ ^(solid diamond), and EEDDE with EGTA (open triangle) or Ca^2+ ^(solid triangle) are shown as examples.

At 25°C the far-UV CD spectra for all of the variants displayed a single negative maximum at ~216 nm, nearly identical to that of wild-type CD2, indicating the maintenance of wild-type β-sheet secondary structure. Moreover, the Trp fluorescence emission spectra of the variants displayed a single maximum at 327 nm, identical to that of the wild-type protein, suggesting that the mutations did not alter the native fold. The CD and the Trp fluorescence spectra remained unchanged with the addition of cations, suggesting the absence of global conformational changes upon metal binding (Fig. 2).

### 3.3. Metal binding

**Figure 3:**
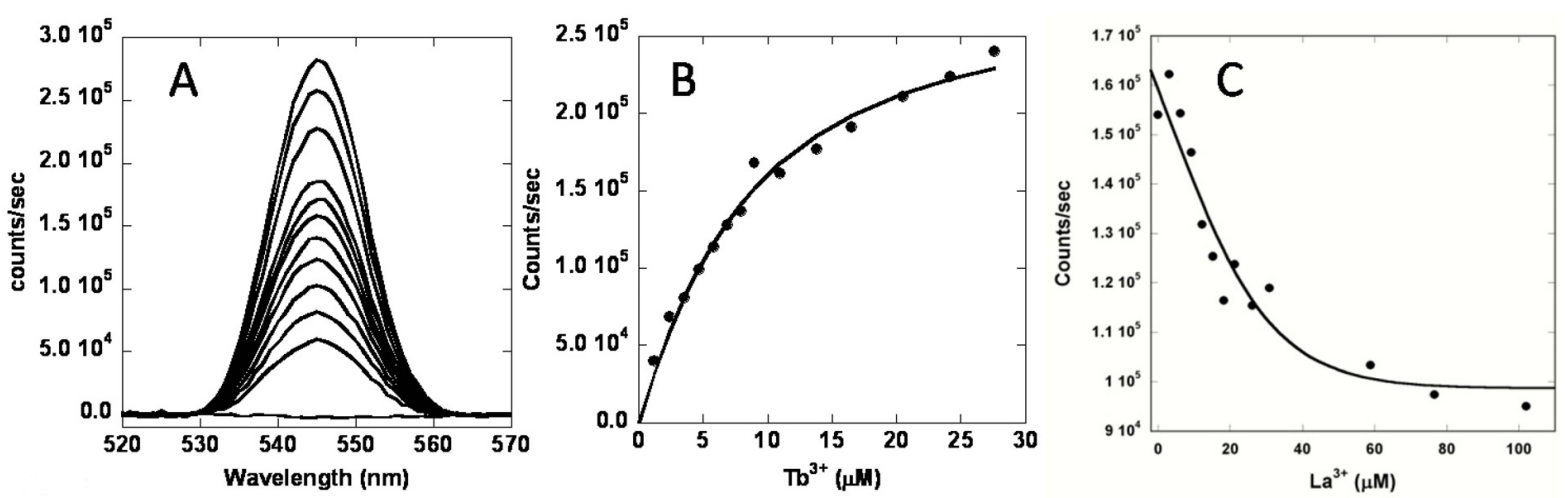
**Metal binding studied by Tb^3+^-FRET**. (A) The fluorescence enhancement at 545 nm with increasing concentrations of Tb^3+ ^in 5 μM of EEDDN in 20 mM PIPES, 10 mM KCl pH 6.8. (B) Determination of Tb^3+ ^binding affinity of EEDDN by analyzing the dependence of the fluorescent intensity change on Tb^3+ ^concentration. The solid line is the fitted curve. (C) Decrease in fluorescence by the addition of La^3+ ^into a fixed concentration of Tb^3+ ^and EEDDN. This data was analyzed to obtain *K*_d _for La^3+^.

Taking advantage of aromatic residues such as Trp-32 in the host protein, we used aromatic sensitized fluorescent resonance energy transfer (FRET) to analyze metal binding in the EEDDD variants (Fig. 3). As negative control, the addition of wild-type CD2 into a Tb^3+ ^solution resulted in negligible change in the Tb^3+ ^fluorescence emission at 545 nm. In contrast, the addition of EEDDD, EEDDE, EEDDN, and EEDDQ into the Tb^3+ ^solution resulted in more than 30-fold increases in Tb^3+ ^fluorescence emission. The addition of NENDN only resulted in a slight enhancement relative to CD2, suggesting that the variant bound Tb^3+ ^at most with a weak affinity. The Tb^3+^-binding affinities were obtained by directly titrating Tb^3+ ^into the protein variants (Table 1). The variants with -5 charges showed the strongest affinities with *K*_d _< 1 μM. The variants with -4 charges show affinities at the μM level. The Tb^3+ ^binding affinity of EENDN was not accurately measured due to the limitation of solubility of the protein in high concentration of Tb^3+^. La^3+ ^binding affinities were obtained by competitively titrating La^3+ ^into Tb^3+^-protein mixtures and monitoring the decrease in Tb^3+ ^fluorescence intensity. The trend of La^3+^-binding affinities for the 7E15 variants was the same as that for Tb^3+^. That is, the -5-charged variants showed the strongest affinities, followed by the -4, -3 and -2 charged variants. The addition of Ca^2+ ^into Tb^3+^-protein mixtures led to only a small decrease in the Tb^3+ ^fluorescence, suggesting inefficient competition by Ca^2+ ^due to weaker Ca^2+ ^binding.

**Figure 4:**
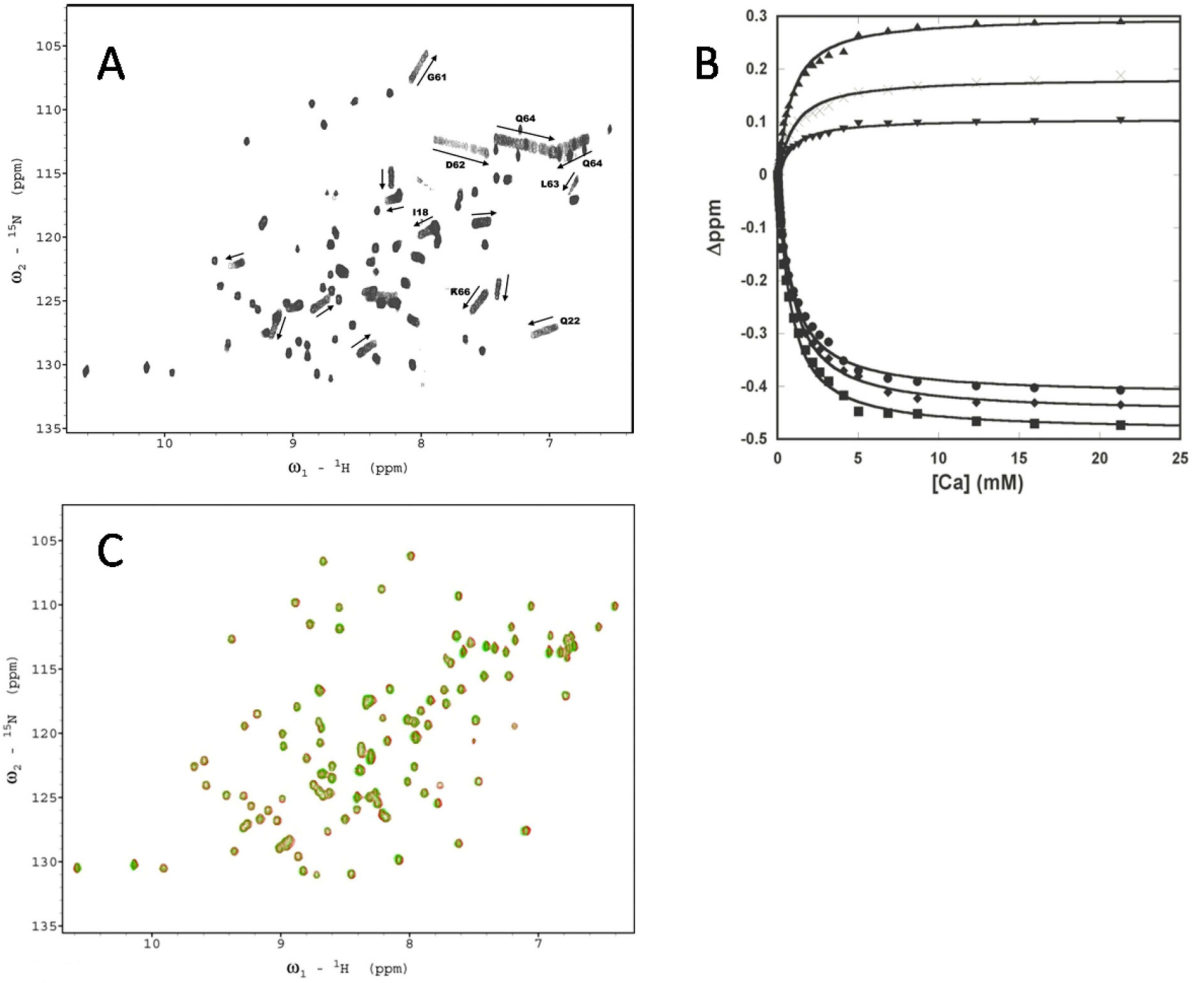
**Metal binding studied by 2D-NMR**. (A) The ^1^H-^15^N HSQC spectra of EEDDQ at different Ca^2+ ^concentrations. The Ca^2+^-induced chemical shift perturbations are indicated by the arrows. Several assigned resonances are labeled. (B) Determination of the Ca^2+^-binding affinity of EEDDQ by following the chemical shift perturbations as a function of Ca^2+ ^concentration. D62 (solid circle); downfield resonance of Q64 sidechain amide (solid square); upfield resonance of Q64 sidechain amide (solid triangle); K66; ×, Q22 (inverted solid triangle). The solid lines are generated by assuming a 1:1 Ca^2+ ^binding to EEDDQ. The results from different resonances are similar. (C) Overlay of the HSQC spectra of NENDN in the presence of 0.05 mM EGTA (red) or 13.1 mM Ca^2+ ^(green).

The Ca^2+^-binding affinity of EEDDD was previously determined to be 100 ± 50 μM from the chemical shift changes of the residues proximate to the metal binding position using ^1^H-^15^N HSQC spectra of ^15^N labeled proteins [32]. In this study, similar Ca^2+ ^titrations on the other variants were performed. The resonances in the spectra for the other variants were partially identified by comparing them to the spectrum of EEDDD. For the -4 and -5 charged variants, Ca^2+ ^specifically perturbed several resonances while other resonances maintained their positions throughout the titration process. Fig. 4A shows the ^1^H-^15^N HSQC spectra of EEDDQ as an example of data obtained. Perturbed resonances were assigned to coordination residues or their neighbours, including G61, L63, Q64, E58, E56, K66, and Q22. The Ca^2+^-binding affinities of EEDDE, EEDDQ, and EEDDN were obtained by analyzing the chemical shift perturbations (Fig. 4B and Table 1). Unlike Tb^3+ ^and La^3+ ^binding, the -4 charged EEDDQ showed a stronger Ca^2+ ^binding affinity than the -5 charged EEDDE. Additionally, the chemical shift perturbations induced by Ca^2+ ^binding to EEDDQ were greater than those to EEDDE. The -3 charged variant EENDN displayed small but significant chemical shift perturbations at high Ca^2+ ^concentrations, while the -2 charged variant NENDN did not undergo any significant changes with the addition of up to 13 mM Ca^2+ ^(Fig. 4C). Hence EENDN possessed only weak Ca^2+ ^binding ability, while NENDN did not show observable Ca^2+ ^binding. The null binding results of EENDN and NENDN confirmed that the chemical shift perturbations observed on the other variants were due to specific Ca^2+ ^binding and not to other nonspecific processes such as salt effects.

### 3.4. Thermal denaturation of 7E15 variants

**Figure 5:**
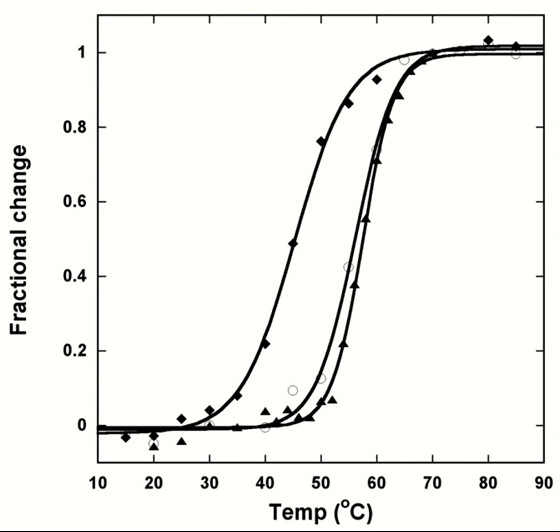
**Thermal denaturation**. The thermal denaturation of EEDDE in the presence of 1 mM EGTA (solid diamond), 10 mM Ca^2+ ^(open circle), or 0.05 mM Tb^3+ ^(solid triangle) in 10 mM Tris, 10 mM KCl. The solid lines are generated by fitting the data to Equation 1.

The thermal transition temperatures (*T*_m_) of all the variants were obtained using far-UV CD (Fig. 5). At 90°C, EEDDD and the other variants were found to be fully unfolded, just like wild type CD2. Compared with CD2, which possesses a *T*_m _of 61 ± 1°C [19], the clustered negative charges at the β-strands decreased the *T*_m_s of EEDDD (41 ± 1°C) and EEDDE (45 ± 3°C) in the absence of metal. Under the same conditions, the *T*_m_s of the -4 charged variants were significantly higher, with values of 53 ± 2°C for EEDDN and 56 ± 2°C for EEDDQ. In the absence of metal, *T*_m _values of EENDN (61 ± 1°C) and NENDN (62 ± 1°C) were similar to that of wild-type CD2 (Table 2).

**Table 2:** *T*_m _values of 7E15 variants

Protein	*T*_m _(°C)		
	
	EGTA	Ca^2+^	Tb^3+^
CD2	61 ± 1	61 ± 1	-
EEDDD	41 ± 1	51 ± 1	-
EEDDE	44 ± 2	56 ± 1	63 ± 2
EEDDN	52 ± 2	58 ± 2	54 ± 1
EEDDQ	56 ± 1	61 ± 1	60 ± 1
EENDN	61 ± 2	59 ± 2	59 ± 2
NENDN	60 ± 3	58 ± 2	58 ± 4

Using far-UV CD all *T*_m _values were obtained in 10 μM protein solution with 1 mM EGTA, 10 mM Ca^2+^, and 0.1 mM Tb^3+^, respectively. The buffer system is 10 mM Tris, 10 mM KCl, and pH 7.4.

A consequence of the assumption that metal ions can only bind to the folded proteins is that the folding stability would be increased by metal binding. Upon binding of Ca^2+ ^or Tb^3+^, the *T*_m _values of the -4 and -5 charged variants indeed increased significantly (Table 2). Ca^2+^-binding increased the *T*_m _of -5 variants EEDDD and EEDDE by ~10°C and Tb^3+ ^binding increased the *T*_m _of EEDDE by ~20°C. Additionally, Ca^2+ ^binding increased *T*_m_of -4 variants EEDDN and EEDDQ by ~5°C, and Tb^3+ ^binding increases the *T*_m _of both variants by ~2-4°C. Neither Ca^2+ ^nor Tb^3+ ^was observed to cause significant changes in the *T*_m _values of EENDN and NENDN, consistent with the weak or non-observable metal binding of these variants.

The order of *T*_m_s of the variants in the absence of metal binding can be summarized as follows: CD2 (-1) ~NENDN (-2) ~EENDN (-3) > EEDDQ (-4) > EEDDN (-4) > EEDDE (-5) > EEDDD (-5). Upon binding Ca^2+^, the order *T*_m_s was largely the same, except that *T*_m _values of EEDDQ and EENDN were the same. Tb^3+ ^binding introduced another alteration in the ordering of *T*_m_s: the *T*_m _of EEDDE now exceeded that of EEDDQ.

### 3.5. Electrostatics calculations

Upon folding, a cluster of like charges is expected to decrease protein stability due to the individual loss of solvation and the collective repulsion among the charges. Upon metal ion binding, the protein and metal ion simultaneously experience unfavorable desolvation and favorable charge-charge attraction. These electrostatic effects were modeled using the Poisson-Boltzmann (PB) equation, from which the electrostatic contributions to folding stability and binding free energy were calculated. Since the CD, fluorescence, and NMR results all showed that the overall tertiary and secondary structures of the variants were similar to those of wild-type CD2, the structures of the variants on the structure of wild-type CD2 were modeled with minimal changes (i.e., all residues other than the one under mutation were fixed). In addition, it was assumed that the metal binding did not perturb the binding pocket or the global conformation of the protein. The calculations of mutational effects on the folding (binding) free energy ΔΔ*G*_f _(ΔΔ*G*_b_) are described in Materials and Methods.

**Figure 6:**
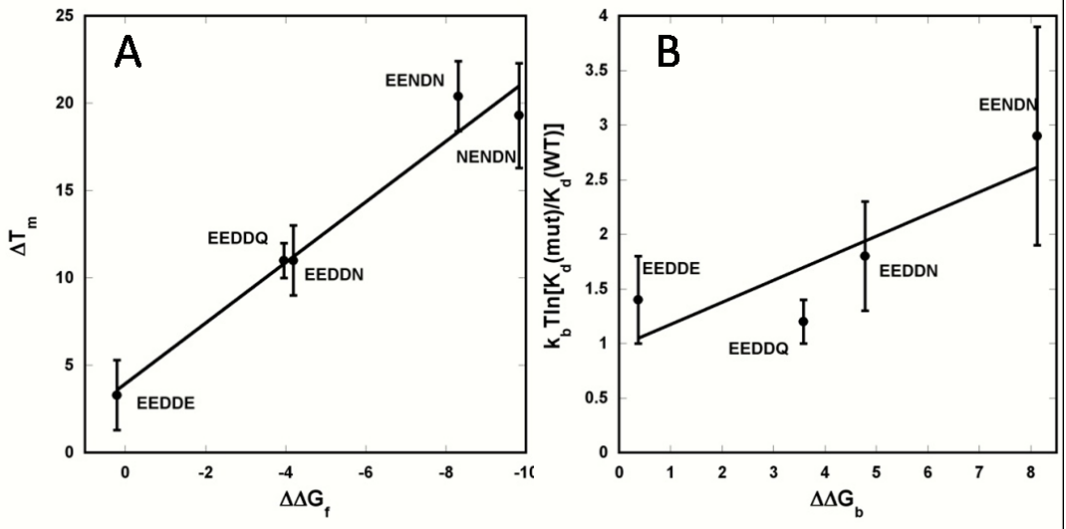
**Electrostatics calculations**. (A) Correlation of observed *T*_m _values of the apo-7E15 variants and calculated changes in folding energy. (B) Correlation of calculated (ΔΔGb) and experimental results for mutational effects on binding free energy. The error bars are for experimental measurements.

The calculated results for ΔΔ*G*_f _correlated well with the experimentally observed *T*_m_s of the apo forms, with *R*^2 ^= 0.91 (Fig. 6A). Both the calculations and the experimental data indicated that the folding stability of the variants is ordered in the following way: EEDDE ~EEDDD < EEDDQ ~EEDDN < EENDN ~NENDN. Apparently, the variants with lower net charges experienced less charge repulsion around the binding pocket, thereby increasing folding stability and *T*_m_.

According to our electrostatic calculations of ΔΔ*G*_b_, the Ca^2+ ^binding affinities of the variants were ordered as follows: EEDDD > EEDDE > EEDDQ > EEDDN > EENDN > NENDN. That is, binding is more favorable at sites with more negative net charge. Compared with the experimental measurements, the order in the Ca^2+ ^binding affinities of EEDDE and EEDDQ was reversed, which may be an indication of non-electrostatic effects not included in our modeling. Nevertheless, there appeared to be good overall agreement between the electrostatic calculations and the experimental measurements regarding the mutational effects on metal-binding affinity (Fig. 6B), suggesting that electrostatic interactions indeed play a major role in the Ca^2+ ^binding affinities of these protein variants.

## 4. Discussion

It is clear that electrostatic interactions play important roles in protein folding and in metal ion binding affinity. As shown in Table 1, trivalent cations bind more strongly to all of the EEDDD variants than divalent Ca^2+^. Moreover, the variants with five negatively charged residues in the metal binding pocket have higher metal ion binding affinities than variants with fewer negative coordination residues. These observations can be simply explained by charge-charge attraction. However, in addition to the direct charge effect, there are multiple factors contributing to the selectivity of different cations, such as coordination properties, binding pocket size, and solvation energy. For example, Zn^2+ ^and Fe^3+ ^prefer sulfur as coordination atoms instead of oxygen [37,38]. Improper pocket size, in addition to influencing cation selectivity, may also alter the van der Waals interaction energy. Ion solvation energy is also an important factor in cation binding and is responsible for the selectivity of Ca^2+ ^over Mg^2+ ^in metalloproteins [37,38]. We have previously shown that designed Ca^2+^-binding sites in proteins exhibit strong metal selectivity for Ca^2+ ^and Ln^3+ ^over excess Mg^2+^, Zn^2+^, Cu^2+^, K^+^, and Na^+ ^[8,19,32,33].

Our highly negative charged designed variants, EEDDD and EEDDE, show the highest binding affinity for metal ions but the lowest folding stability. Binding is more favorable in EEDDD than EEDDE, most likely due to the extra methylene group in the latter, which has the effect of reducing the local charge density, hence decreasing cation binding affinity. This extra methylene group also appears to influence protein stability, as the *T*_m _comparisons of EEDDD/EEDDE and EEDDN/EEDDQ suggest that mutation of Lys 64 by Glu instead of Asp or by Gln instead of Asn increases the thermal stability for both apo and loaded forms (Table 2). It is plausible that the extra methylene group in the carboxylic sidechain reduces the local repulsion among charged coordination residues. A statistical study has shown that Asp is the most preferred coordination residue in naturally-evolved Ca^2+ ^binding sites followed by Glu, Asn, and Gln [16,39]. That is, charged coordination residues are preferred over neutral ones (D and E over N and Q) due to the charged nature of Ca^2+^. The calculation and experimental results reported here for the EEDDD variants indicate that steric size of the coordination residues is a less important factor for Ca^2+ ^binding.

The introduction of negative charges and the removal of positively charged K64 in all of the EEDDD variants disrupt the local charge balance of CD2 (Fig. 1). Binding of Ln^3+ ^or Ca^2+ ^neutralizes the charge repulsion, resulting in an increase in the thermal transition temperature. The increase in *T*_m_, = 10°C, is more dramatic for the most negatively charged variants (EEDDD and EEDDE). Based on the binding free energy calculations, the effects of cation binding on the *T*_m _can be inferred. Weaker binding, as indicated by a positive ΔΔ*G*_b_, is expected to correspond to a smaller increase in *T*_m_. The small ΔΔ*G*_b _value calculated for EEDDE (0.4 kcal/mol) in reference to EEDDD suggests that in the presence of cation binding, EEDDE shows an increase in *T*_m _as significant as EEDDD. The moderate ΔΔ*G*_b _values calculated for EEDDQ (3.6 kcal/mol) and EEDDN (4.8 kcal/mol) suggest that these two variants show a moderate increase in *T*_m_. On the other hand, the relatively large ΔΔ*G*_b _values of EENDN (8.2 kcal/mol) and NENDN (10.2 kcal/mol) suggest a relatively small increase in *T*_m _upon cation binding. These predictions agree well with the experimental results.

The predictions of mutational effects by our electrostatic calculations, both on folding stability and on cation binding affinity, achieved good correlations with the experimental results. However, the predictions differed from the experimental results in magnitudes, due to the simplicity of our electrostatic model. First of all, we used fixed charges. In reality, charges will redistribute due to electronic polarization upon folding or upon cation binding; the use of fixed charges will exaggerate electrostatic contributions. Such exaggeration is particularly severe for cation binding, since the residues under mutations are directly coordinated with the bound ion. Second, in modeling mutations, residues other than the one under mutation were fixed. In reality, surrounding residues will readjust; such relaxation will mitigate the mutational effect. Third, the electrostatic calculations were carried out on a single protein conformation. Inclusion of conformational sampling (e.g. by molecular dynamics simulation) as opposed to single conformation calculation, appears to increase the predictive power of our model [40,41]. Finally, we totally neglected non-electrostatic effects, such as hydrophobic and van der Waals interactions.

In summary, using *de novo *designed Ca^2+ ^binding proteins, which circumvent complications related to cooperative binding and Ca^2+^-induced conformational change in natural proteins, enabled us to reach several conclusions. By increasing the number of negatively charged coordination residues from 2 to 5 in a relatively restricted Ca^2+^-binding site, the Ca^2+ ^binding affinities were increased by more than 3 orders of magnitude. The metal selectivity for trivalent Tb^3+ ^over divalent Ca^2+ ^was also increased by more than 100 fold. On the other hand, increasing the number of negatively charged coordination residues decreased the thermal transition temperatures of the apo-proteins due to the repulsion between the negatively charged residues in the apo-form. The thermal stability of the proteins was regained upon Ca^2+ ^and Ln^3+ ^binding to the designed Ca^2+ ^binding pocket. Our study thus show that the charged coordination residues increase Ca^2+ ^and Ln^3+ ^affinity at the expense of decreased protein stability due to the charge repulsion of the Ca^2+^-free form. Furthermore, charge numbers of -3 or -4 for Ca^2+ ^binding favor protein stability as well as Ca^2+ ^binding. The steric size of the coordination residues is not crucial as long as the Ca^2+^-binding pocket is properly formed. In addition to revealing key factors involved in Ca^2+ ^binding affinity and Ca^2+^-conferred thermal stabilization in natural Ca^2+ ^binding proteins, our results regarding the net charge and coordination type provides important insights into engineering proteins such as thermoenzymes with enhanced stability [42-44].
